# Fibrosarcomatous Dermatofibrosarcoma Protuberans of the Head

**DOI:** 10.7759/cureus.60110

**Published:** 2024-05-11

**Authors:** Renato C Galvan, Paul Vincent Opinaldo

**Affiliations:** 1 Neurology, Quirino Memorial Medical Center, Quezon City, PHL; 2 Neurology, St. Lukes Medical Center, Quezon City, PHL

**Keywords:** sma, cd34+, imatinib, fibrosarcomatous transformation, dermatofibrosarcoma protuberans

## Abstract

This article highlights the case of a 37-year-old male who presented with a recurrent, exponentially enlarging head mass, emphasizing the diagnostic and therapeutic challenges associated with a very rare type of tumor, fibrosarcomatous dermatofibrosarcoma protuberans (DFSP) of the head. Our patient presented with a rapidly growing head mass, initially diagnosed as a spindle cell tumor, and was managed with surgical excision and skin flap grafting. Follow-up revealed relapse and interval development of hemiparesis and hemisensory loss. MRI revealed tumor recurrence, with compression of the right parietal lobe and superior sagittal sinus. Histopathology revealed stroma with fascicles of spindle cells homogenous to fibrillar cytoplasm, with oval vesicular nuclei. Immunohistochemical staining showed positivity for CD34 and SMA. Oral chemotherapy with imatinib 800 mg/day was started. Follow-up imaging showed a marked reduction in the size of the tumor and resolution of the compression of the underlying brain parenchyma with cystic degeneration and decreased contrast enhancement. Future plans include possible surgical tumor debulking and/or radiation therapy. Although extremely rare, awareness of this tumor, with a multi-disciplinary approach to the management of the case, is vital to maximize treatment outcomes.

## Introduction

Dermatofibrosarcoma protuberans (DFSP) is a rare type of sarcoma that arises from the fibroblasts and tends to become locally aggressive [1]. The tumor begins as a gradually enlarging palpable lesion over the skin, mostly affecting the trunk and extremities. Despite their rarity, cases involving the head and neck, including the face and scalp, have been described [2].

In the 2020 WHO Classification of Soft Tissue Tumors, the neoplasm was reclassified from low to intermediate type of fibroblastic tumor, representing less than 10% of all known soft tissue sarcomas [3]. In the Philippines, DFSP accounts for less than 3% of all skin and soft tissue tumors seen at the Philippine General Hospital [4].

Fibrosarcomatous transformation, an infrequent variant and the lone subtype of DFSP cited as clinically significant, features potential for invasion, recurrence, and metastatic spread, particularly to the lungs [5]. Interestingly, fibrosarcomatous DFSP of the head has only been recorded sparingly in literature [6].

Grossly, DFSP is seen as an enlarging mass extruding from the skin. Microscopically, it is comprised of homogenous, spindle-shaped cells, in a storiform pattern, showing strong and diffuse positivity to CD34 on immunohistochemistry.

The cause of the tumor is unknown, but a chromosomal translocation that produces a fusion protein leading to the overproduction of platelet-derived growth factor (PDGF) and upregulation of its receptor has been implicated [7]. Imatinib, either as an adjuvant or neoadjuvant agent to maximal surgical excision and/or radiation therapy, inhibits this receptor.

## Case presentation

A 37-year-old Filipino male with a two-month history of head trauma presents to the institution for a gradually enlarging head mass over the midparietal region, associated with mild headache. Past medical, family, and personal social history were unremarkable. There was no exposure to any heavy metal or industrial chemicals. On examination, an irregularly bordered, non-tender, firm, fix-based growth was appreciated. MRI revealed a large lobulated soft tissue outwardly expanding mass over the right parietal scalp, measuring 8.0x8.2x7.7 cm (APxTxCC), with thinning and disruption of the outer table of the underlying parietal bone, but with no intracranial extension. Excision of the tumor with a wide surgical margin was done. Biopsy sections showed tissues lined by keratinizing stratified squamous epithelium and stroma with fascicles of plump spindle cells with homogenous to fibrillar cytoplasm, with oval vesicular nuclei, with evidence of five to ten mitoses per high power field. Histopathologic diagnosis was spindle cell neoplasm favoring intermediate grade. Immunohistochemical stains were suggested but due to financial constraints were not facilitated. The post-surgical site showed no residual tumor cells on histopathology, and hence, skin flap grafting was completed. No further metastatic work-up was done at this time. The patient was discharged on improvement but, thereafter, was lost to follow-up.

**Figure 1:**
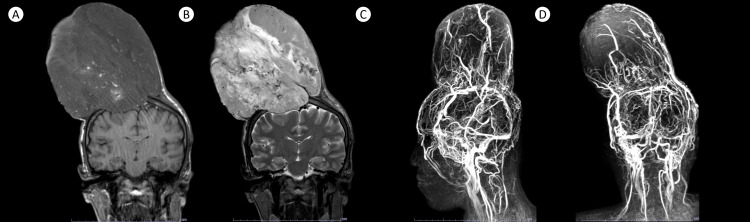
Representative images of the patient’s MRI show a large heterogeneously enhancing tumor, measuring 14.5x14.2x11.9 cm (APxTxCC), shown in coronal T1-weighted (A), T2-weighted (B), sagittal (C), and coronal (D) images. MRV images show a highly vascular tumor that receives supply from scalp arteries and drains via cortical and emissary veins.

Three years post-operatively, the patient had inadvertent trauma over the post-surgical site. He presents with a new-onset, exponentially expanding mass over the area, associated with moderate headache, left-sided hemiparesis and hemisensory loss, and limitations in activities of daily living. Excoriated lesions on the tumoral surface caused bleeding and local areas of infection. Repeat MRI showed a large enhancing heterogeneous soft tissue mass ascribed to tumor recurrence with cystic components representing necrosis and susceptibility artifacts denoting hemorrhage and calcifications. Concomitant compression of the right frontoparietal lobes and superior sagittal sinus was evident (Figure 1). A metastatic work-up revealed probable metastasis to the right lower lung.

**Figure 2:**
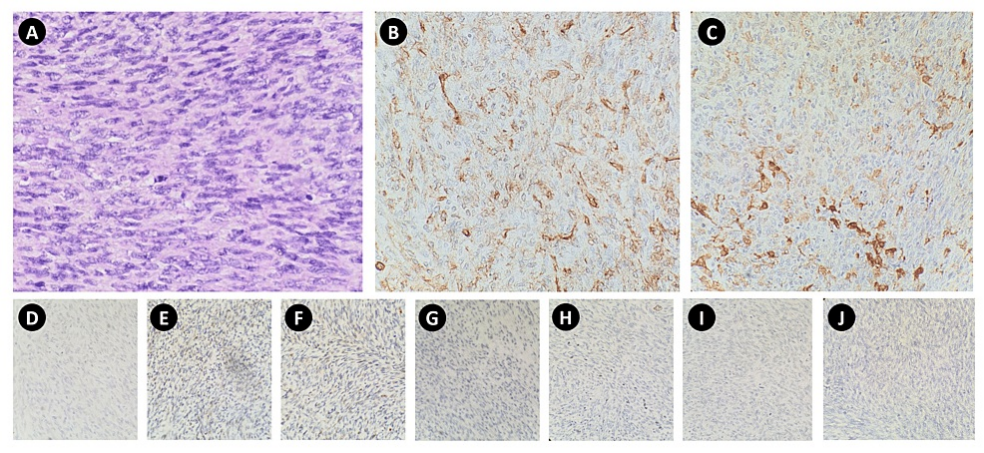
Representative slides of the tumor show spindle-shaped cells with homogeneous fibrillar cytoplasm and oval vesicular nuclei in HE stain (A). Cells were diffusely positive for CD34 (B), focally positive for SMA (C), negative for desmin (D), cytokeratin (E), S100 (F), STAT6 (G), TLE1 (H), MUC4 (I), and EMA (J).

Immunohistochemical staining was pursued and showed diffuse positivity for CD34 and focal positivity SMA, but was negative for desmin, cytokeratin, S100, STAT6, TLE1, MUC4, and EMA (Figure 2). The report was signed out as DFSP with fibrosarcomatous transformation. Molecular testing for COL1A1-PDGFB rearrangement was suggested.

**Figure 3:**
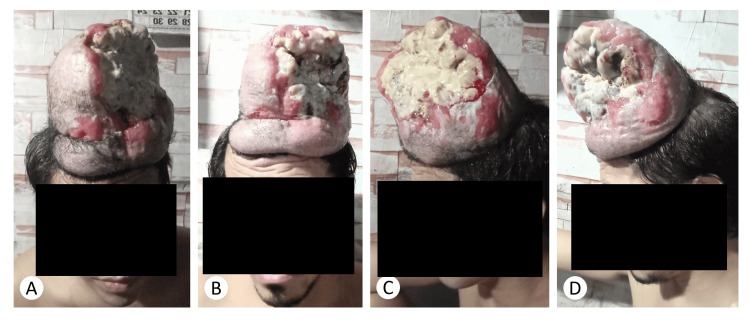
Actual photographic documentation of the reduction of the tumoral size at baseline (A,C) in comparison with four months post-imatinib (B,D).

The patient was started on imatinib, initially at 400 mg/day, but later progressed to 800 mg/day to maximize response. No adverse effects were noted. Grossly, after treatment, there was an evident reduction in tumor size and areas of necrosis (Figure 3).

**Figure 4:**
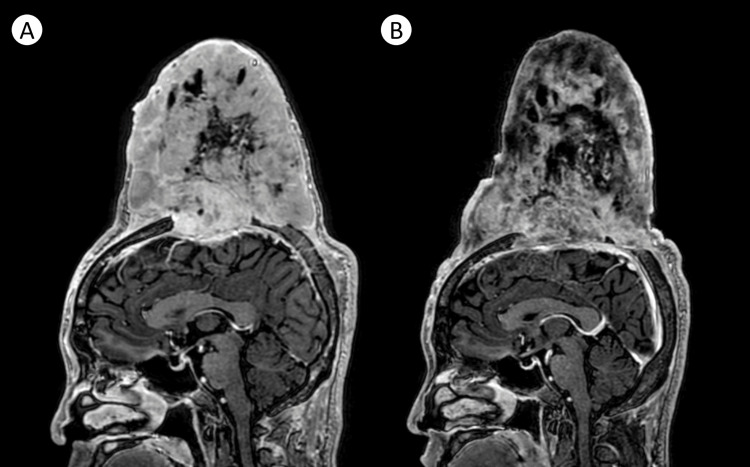
Representative sagittal T1-weighted MRI images of the tumor prior to treatment (A) and four months post-imatinib (B), showing an overall reduction in tumoral volume and size, now measuring 12.4x12.0x14.3 cm (APxTxCC), with decreased enhancement cystic degeneration, attributable to tumor necrosis.

A repeat MRI done four months post-therapy showed a decrease in the overall size, volume, and contrast enhancement of the tumor with a decrease in the degree of compression of the underlying frontoparietal lobe (Figure 4). Further treatment plans include possible surgical tumor debulking and possible radiation therapy.

## Discussion

First described by Hoffman in 1925, DFSP represents an enigma, in that it presents both diagnostic and therapeutic dilemmas for the clinician. Worldwide, the tumor affects males in the third to fourth decade of life, particularly those of African descent. In the Philippines, profiling done by Villaneuva in 2022 showed that the tumor affects middle-aged adults with a median age of 31, with a 67 in 100 male-to-female ratio [4]. Risk factors include trauma, the existence of preexisting scars, and tattoos.

The majority of DFSP have a translocation, t(17;22) (q22;q13), allowing a constitutionally inhibited platelet-derived growth factor β (PDGFβ) gene under the activating control of the collagen type 1 α 1 (COL1A1) promoter, resulting in overproduction of the PDGFβ polypeptide and constitutive activation of PDGFβ tyrosine kinase receptor, leading to neoplastic growth [7]. Such genetic signature is best detected through FISH assays and multiplex RT-PCR.

DFSP is primarily exophytic, consisting grossly of poorly circumscribed lesions that can gradually but progressively enlarge [8]. Morphologically, DFSP classically presents with homogenously appearing bundles of spindle cells, arranged in a storiform pattern. On immunohistochemistry, DFSP shows strong and diffuse positive staining for CD34 while being negative for factor XIIIA, desmin, D2-40, or CD163 (markers more commonly seen in benign dermatofibroma). Features of rare variants and pitfalls to their diagnosis have been characterized in full by Trinidad et al. in 2023 [5].

Fibrosarcomatous transformation of DFSP is seen in less than a quarter of all cases; has been described to be locally aggressive; has a high propensity to recur; and capacitates metastatic spread due to its increased cellularity, high mitotic activity, and deep tissue involvement [9]. Although the mechanism for fibrosarcomatous transformation has not been fully elucidated, this process is considered a form of tumor progression.

Head DFSP, which accounts for less than 10% of all DFSP cases, can be challenging to recognize and manage. In the series by Dai et al. (2023), the similarity of the initial presentation of DFSP with other lesions of the head such as solitary fibrous tumors, peripheral nerve sheath tumors, or benign fibrosarcomas predisposes to risk of delays or errors in diagnosis, hence, the emphasis on the accurate histomorphological description of the biopsy and adequate immunohistochemistry [1].

Fibrosarcomatous DFSP of the head, particularly those involving the scalp, represents the farthest end of the spectrum of DFSP. Left untreated, the aggressive behavior of the tumor can jeopardize underlying neurovascular structures [9]. In the cohort analysis by Go et al. (2022), factors such as advanced age, tumor size, and socioeconomic status are the most significant predictors of survival in head DFSP [3]. 

Currently, no standard staging system for head DFSP is available, but a modified staging system was proposed by Hao et al., based on the European consensus-based interdisciplinary guideline in 2015 [8].

MRI remains the radiologic imaging of choice for DFSP. Accurate anatomical delineation of the tumor is vital in treatment planning. More recently, high-resolution dynamic contrast-enhanced MRI has been described to be of clinical usefulness, particularly in identifying tumoral infiltration and invasive growth [10].

Maximal surgical excision with a clear wide tumor-free margin of at least 3 cm remains the cornerstone of treatment of DFSP, as this reduces the risk of local invasion or metastasis [11]. Mohs micrographic surgery with smaller lesions may be appropriate. In head DFSP, cosmetic consequences of treatment are given consideration, hence, warranting a multi-disciplinary approach to treatment. Reconstruction techniques may be required, should the tumor involve the face. In rare cases of DFSP invading the skull bone, reconstruction using muscle and skin flaps may be necessary [12]. More advanced centers have also used acellular dermal matrix (ADM) for scalp reconstruction [13].

Imatinib, a selective tyrosine kinase receptor inhibitor, is useful as an adjuvant therapy for patients deemed unfit for surgery due to the extent of the disease or the possible functional and cosmetic consequences of the operation [14]. Imatinib shows promise as a neoadjuvant agent, leading to median tumor volume shrinkage of 20-30%. Locally advanced and metastatic DFSP also showed good response, with less than 10% showing progression and/or recurrence. Adverse effects mostly involved dermatologic reactions. Despite the number of studies highlighting its utility, the duration of treatment, timing of discontinuation, as well as resistance to imatinib treatment still warrant further investigation.

Radiotherapy remains a controversial modality of treatment for DFSP but is an option for patients with indeterminate or positive surgical margins. Local tumoral invasion and poor surgical candidates may require post-operative radiotherapy for better control and reduced risk of recurrence. A dose of 60-70 Gy (given 2 Gy daily, five times weekly), for indeterminate and positive margins, respectively, have been described [15].

Head DFSP carries a good prognosis, provided the tumor is removed in toto, with more than 90% ten-year survival. Lung and bone metastases dictate poorer outcomes. Regular, periodic follow-ups of patients to clinically assess post-operative sites with an active search for possible local invasion and/or distant metastasis are crucial [1,2].

## Conclusions

Fibrosarcomatous DFSP of the head is an extremely rare malignant tumor with invasive, recurrent, and metastatic potential. In our case, recurrent trauma and prior surgical scars were predisposing factors. Gross, histomorphological, immunohistochemical, and radiological characteristics clinched the diagnosis. Imatinib proved efficacious. Surgical and radiation oncology planning continues for the patient. Subjectively, the patient reports improvement in overall quality of life. Limitations include genetic evaluation for COL1A1-PDGFB gene rearrangement, which is not locally available. This article was previously posted to the Research Square preprint server on September 11, 2023.
